# Antibodies to *S*. *aureus* LukS-PV Attenuated Subunit Vaccine Neutralize a Broad Spectrum of Canonical and Non-Canonical Bicomponent Leukotoxin Pairs

**DOI:** 10.1371/journal.pone.0137874

**Published:** 2015-09-14

**Authors:** Rajan P. Adhikari, Thomas Kort, Sergey Shulenin, Tulasikumari Kanipakala, Nader Ganjbaksh, Mary-Claire Roghmann, Frederick W. Holtsberg, M. Javad Aman

**Affiliations:** 1 Integrated Biotherapeutics Inc., Gaithersburg, Maryland, United States of America; 2 Department of Epidemiology and Public Health, University of Maryland School of Medicine, Baltimore, Maryland, United States of America; 3 VA Maryland Health Care System, Baltimore, Maryland, United States of America; Indian Institute of Science, INDIA

## Abstract

*S*. *aureus* vaccine development has proven particularly difficult. The conventional approach to achieve sterile immunity through opsonophagocytic killing has been largely unsuccessful. *S*. *aureus* is highly toxigenic and a great body of evidence suggests that a successful future vaccine for this organism should target extracellular toxins which are responsible for host tissue destruction and immunosuppression. Major staphylococcal toxins are alpha toxin (a single subunit hemolysin) along with a group of bicomponent pore-forming toxins (BCPFT), namely Panton-Valentine leukocidin (PVL), gamma hemolysins (HlgCB and AB), LukAB and LukED. In our previous report, an attenuated mutant of LukS-PV (PVL- S subunit) named as “LukS-mut9” elicited high immunogenic response as well as provided a significant protection in a mouse sepsis model. Recent discovery of PVL receptors shows that mice lack receptors for this toxin, thus the reported protection of mice with the PVL vaccine may relate to cross protective responses against other homologous toxins. This manuscript addresses this issue by demonstrating that polyclonal antibody generated by LukS-mut9 can neutralize other canonical and non-canonical leukotoxin pairs. In this report, we also demonstrated that several potent toxins can be created by non-canonical pairing of subunits. Out of 5 pairs of canonical and 8 pairs of non-canonical toxins tested, anti-LukS-mut9 polyclonal antibodies neutralized all except for LukAB. We also studied the potential hemolytic activities of canonical and noncanonical pairs among biocomponent toxins and discovered that a novel non-canonical pair consisting of HlgA and LukD is a highly toxic combination. This pair can lyse RBC from different species including human blood far better than alpha hemolysin. Moreover, to follow-up our last report, we explored the correlation between the levels of pre-existing antibodies to new sets of leukotoxins subunits and clinical outcomes in adult patients with *S*. *aureus* bacteremia. We found that there is an inversed correlation between the antibody titer to sepsis for leukotoxins LukS-mut9, LukF-PV, HlgC, LukE and LukAB, suggesting the risk of sepsis was significantly lower in the patients with higher antibody titer against those toxins.

## Introduction

*Staphylococcus aureus* (SA), a gram positive bacteria, is one of a major cause of hospital-associated (HA) and community-associated (CA) infections worldwide. These infections range from minor skin and soft tissue infections (SSTI) to the major life-threatening invasive infections [1, 2]. Many virulence factors including coagulases, adhesins, proteases and capsular polysaccharides (CP) contribute to infection and disease progression. In addition, exoproteins such as pore-forming toxins and superantigens are equally responsible for the pathology and help the microbe to cope with host’s innate and adaptive immune responses [1, 2]. Recent studies have shown that antibodies to poly-N-acetylglucosamine and capsular polysaccharides (CP) play a negative role by interfering with the protective activity of immune-induced antibodies against *S*. *aureus* capsular polysaccharides, raising a question for successful use of surface antigens to induce sterile immunity [3, 4]. In contrast, conjugating attenuated alpha toxin (dHla) to the CP5 or CP8 vaccine were better in reducing bacterial load and bone morphological changes compared with group immunized with vaccine alone [5]. The iron-responsive surface determinant B (IsdB) vaccine (V710) not only failed in phase II/III clinical trial but also led to high incidence of multiorgan failure in vaccinated groups compared to placebo [6, 7]. These studies suggested that surface antigen based vaccine candidates are not sufficient to generate protective efficacy and may indeed exacerbate the disease and a successful vaccine may require neutralization of key toxins such as superantigens and pore-forming toxins.

The bicomponent pore-forming toxins (BCPFTs) group of toxins consist of “S” and “F” subunits. The S-subunit is the primary receptor binding subunit [8, 9]. The binding of the S- subunit to the primary receptor triggers association with the F subunit and oligomerization of BCPFTs leading to pore formation on polymorphonuclear cells (PMNs), red blood cells (RBCs), macrophages, and lymphocytes with varying cellular tropism between the different BCPFTs [10, 11]. Among these, HlgAB, HlgCB, LukED, LukAB are the members of the leukocidin family which are chromosomally encoded whereas, Pantone-Valentine leukocidin (PVL) is phage-encoded [12–15]. HlgAB and HlgCB are toxic to human and other mammalian RBCs [16] and PMNs [17, 18]. LukED [19] and LukAB [20] play a significant role in SA pathogenesis in mouse models. However, the contribution of PVL as a virulence factor has been a controversial. Some studies have shown protection against while other studies have demonstrated enhancement of infection depending on the model used. It appears that the cytolytic activity of PVL is strictly species specific; the human and rabbit cells are affected by this toxin whereas, mouse and nonhuman primates cells are resistant [21]. We have previously shown that an attenuated LukS-PV subunit vaccine (LukS-mut9) protects mice against SA intraperitoneal infection [22]. However, given the lack of PVL receptor in mice, the observed efficacy may be attributed to cross neutralization of other leukotoxin S subunits with significant homology to LukS-PV. Therefore, we examined the breadth of neutralizing activity of anti-LukS-mut9 polyclonal antibodies against the major leukotoxins in this study. It has also been reported that non-canonical S and F subunits can pair to form functional toxins [23, 24]. Here we further examined the breadth of toxic activities of such non-canonical BCPFTs and examined their neutralization by antibodies to LukS-mut9.

## Materials and Methods

### Plasmids, protein expression and purification

cDNA for wild type leukotoxins: HlgA, LukA, LukB, LukE and LukD were cloned into pET24a (+) vector. For LukS-PV, LukF-PV, HlgB, and HlgC subunits. The cDNAs with an N-terminal 6xHis tag were synthesized and cloned into the pQE30 vector. The generation of LukS-mut9 was described previously by our group [22].

For expression and purification of the recombinant wild type leukotoxins the pET 24a(+) vectors we moved into BL21 (DE3) *E*.*coli* cells (NEB, MA) and the pQE30 vectors into *E*. *co*li XL1-Blue cells. Induction was carried out by the addition of 0.3 mM IPTG to log phase bacterial cultures (0.5–0.6 A_600_) followed by incubation for 2.5 h at 37°C or overnight at 25°C. His-tagged leukotoxin subunits were purified using HisTrap HP columns as described previously [25]. The HlgA, LukE and LukD were purified through a combination of IEX and mixed-mode chromatography, subsequent to nucleic acid removal and ammonium sulfate precipitation. For LukA and LukB, the strategy to individually express the subunits resulted in expression that was completely insoluble, and attempts to refold the subunits separately yielded subunits with low activity. Hence, we developed strategies to individually solubilize, and then refold the subunits together. The refolded, enriched LukAB product, although completely soluble, may not be completely native. Bicinchoninic acid (BCA) assays were used to determine protein concentration. Western blot analysis was performed using rabbit polyclonal antibodies raised against the respective peptide antigens. Molecular weight and protein purity was determined by SDS PAGE for each protein. Chromogenic endotoxin assay (Limulus Amoebocyte Lysate) was used to determine the endotoxin levels in protein samples as described in our previous report [25]. The proteins were aliquoted and stored at − 80°C.

### Enzyme-linked immunosorbant assay (ELISA)

ELISA was used to determine the antibody titer for clinical samples as described in our previous report [26]. Briefly, 96-well plates were coated overnight at 4°C with 100ng/well of various leukotoxin subunits. After blocking with 4% non-fat milk in PBS, dilutions of human serum samples (in 1% non-fat milk in PBS) were applied and plates were incubated for an hour at room temperature. After washing three times, goat anti-human IgG (H&L)-HRP was added in the same buffer. Plates were incubated with conjugates for an hour, washed and incubated for 30 minutes with TMB. Optical density (OD) 650nm was measured in Versamax plate reader (Molecular Devices, CA). Statistical analysis was done by student's unpaired t-test using GraphPad Prism 5 (GraphPad Software).

### Leukotoxin cytotoxicity and neutralization assay

Cytotoxicity of canonical and non-canonical leukotoxin pairs were determined in dimethylsulfoxide (DMSO) induced HL-60 cells (ATCC, Manassas, VA). The cells were cultured for seven days in RPMI media supplemented with 15% fetal bovine serum (FBS) and 1.5% DMSO before the assay. The cells were then harvested and washed with RPMI media with 2% FBS. Cytotoxicity assay was performed as described previously [26]. Rabbit polyclonal anti-LukS-mut9 (1:24 dilution) or naive rabbit IgG was used as a control for neutralization experiments. Antibodies were serially diluted and incubated with a fixed concentration of the toxin for 30 minutes before mixing with HL-60 derived neutrophils at a final density of 5 x 10^5^ cells/well, then incubated for 3 hours at 37°C and 5% CO_2_. This mixture was further incubated with 100 μg/ml of XTT (Sigma-Aldrich, St. Louis MO) for 16 hours and the cell viability was measured. Neutralization was determined in terms of percent cell survival for each sample by colorimetric measurement at OD_470_ nm [26].

### Alpha toxin hemolytic and neutralization (TNA) assay

Blood from different animal species were obtained from Colorado serum company (Denver, CO). Hemolytic assays were carried out as previously published [25]. Briefly, RBCs were purified from blood by centrifugation and re-suspended in PBS to a concentration of 4% (weight/volume). For hemolytic assays different concentrations of the toxins were mixed 1:1 with 4% RBCs in 96-well plates and incubated at 37°C for 30 minutes (for Rabbit, Guinea pig hemolytic assays) or for 45 minutes at 37°C (for human, horse and sheep hemolytic assays). Plates were then centrifuged and 100ul of supernatants were transferred to NUNC plates and hemolysis was measured colorimetrically at OD_416_ nm. The 50% effective concentration (EC50) of alpha toxin was determined by plotting the OD_416_ nm versus hemolysis using a 4PL fit. For TNA, alpha toxin and/or different leukotoxins pairs were incubated at RT for 10 minutes with diluted serum samples, and the mixtures were added to the RBCs as described above. Neutralization was determined based on percent inhibition of hemolysis in the presence of neutralizing serums.

### Mouse HlgA and LukD lethality and LukS-mut9 rescue study

Female ICR mice, 6–8 weeks old, were purchased from Charles River laboratories, housed under pathogen-free conditions, and fed laboratory chow and water ad libitum. The experiments planned involved injecting no more than 100μl intravenously (IV) containing 25 μg of total protein (S and F subunit) for different leukotoxin pairs. A 1 cc syringe (½-1 inch, 25–28 g needle) was used for all injections. Mice were placed underneath a heat lamp for vasodilation and briefly restrained during the IV injection procedure.

For animal rescue experiments, we purified the total antibodies from crude LukS-mut9 serum samples by protein A purification column. Animal experiment was carried out in 3 groups of BALB/c mice (5 mice/group). For PBS group, 100ul of PBS was injected by IV route. For LukS- mut9 immunized group, we immunized mice passively with 750 ug/mouse anti-lukS-mut9 polyclonal (total antibody) 5 minutes before challenge with 10ug/mouse of toxin (HlgA + LukD) in 100 ul volume by IV route. For toxin alone group, 10ug/mouse of toxin (HlgA + LukD) was challenged by IV route in 100 ul volume.

Animal (mouse) work was performed with protocols approved by institutional animal care and use committees (IACUC) of Nobel Life Sciences (NLS) (Gaithersburg, MD 20878) (OLAW registration number is A4633-01). Incoming animals were received from NIH approved animal suppliers only and, were quarantined for a period of approximately 5 days. A single animal technician was responsible for quarantine. Animals were caged in ventilated cages in accordance to the NIH Guidelines.

Mice were monitored for mortality and morbidity every minute for the first hour followed by twice daily monitoring for mortality and morbidity for an additional 3 days following challenge. Any animals displaying severe illness as determined by >30% weight loss, extreme lethargy, or paralysis was euthanized. Mice were euthanized in accordance with the 2007 AVMA Guidelines on Euthanasia and institutional SOPs. Briefly, mice were subjected to CO2 inhalation for at least 4 minutes, using a gas cylinder or a house line, followed by exsanguinations via cardiac puncture, and confirmed by cervical dislocation by qualified NLS personnel. Death was verified by absence of a heart beat at no less than 5 minutes post-exsanguination.

### Serum samples

Samples from patients (adult 18 years and older) whose *S*. *aureus* infections were complicated by bacteremia and hospitalized at 4 hospitals in Maryland from 2009 to 2011 were collected and collection were done prior to (0–3 days before the day the positive blood culture was drawn) or at the time of their bacteremia. Patients were followed-up for clinical outcomes of either the development of sepsis within 3 days of bacteremia, defined as severe sepsis or septic shock [27], or recovery. The study was approved by the UM Baltimore Institutional Review Board and was granted a waiver of informed consent. The detailed collection methods are fully described in our previous report [26].

## Results

### Generation and production of BCPFT subunits

**Fig 1:**
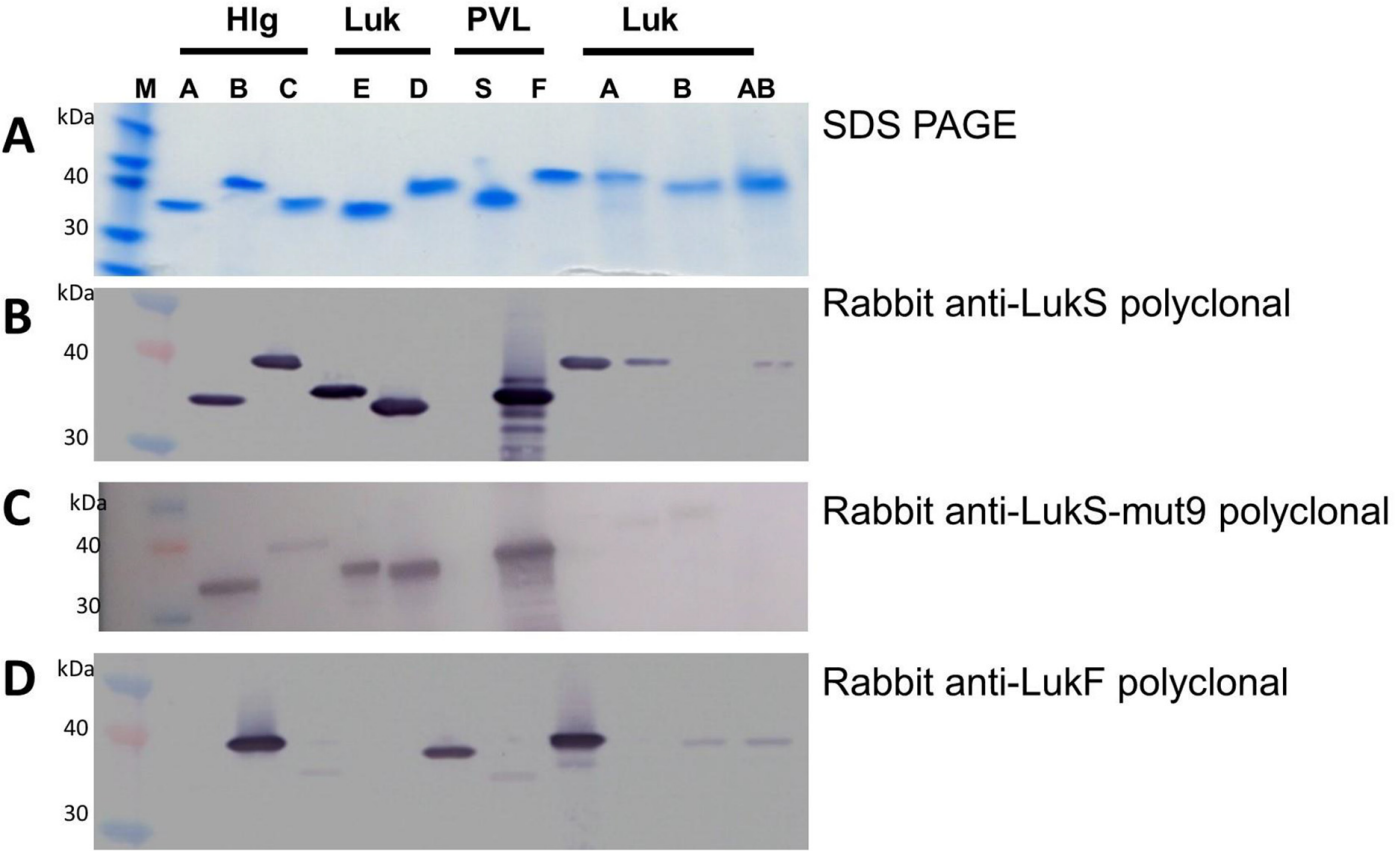
SDS-PAGE and Western blot of different subunits. ***A)*** SDS PAGE, ***B)*** Western blot with Rabbit anti- LukS-WT polyclonal, ***C)*** Western blot with Rabbit anti-LukS-mut9 polyclonal, ***D)*** Western blot with Rabbit anti-LukF-WT polyclonal.

The leukotoxin subunits were purified using HisTrap columns or a multistep chromatography as described in the Materials and Methods section. For LukAB we noted that co-refolding of the subunits lead to a higher yield consistent with a previous report [28]. The BCPFT subunits displayed the expected molecular weight and over 95% purity as shown by SDS-PAGE (**Fig 1A**). Western blots analysis was carried out using rabbit anti- LukS polyclonal (**Fig 1B**), rabbit anti- LukS-mut9 polyclonal (**Fig 1C**) and rabbit anti-LukF polyclonal antibodies (**Fig 1D**). Cross detection of S and F subunits was observed due to their high sequence homology. We also confirmed the identity of each subunit by western blot analysis using affinity purified rabbit polyclonal antibodies generated against specific peptides unique to each subunit (data not shown).

### PMN cytotoxicity of canonical and non-canonical subunit pairs

**Fig 2:**
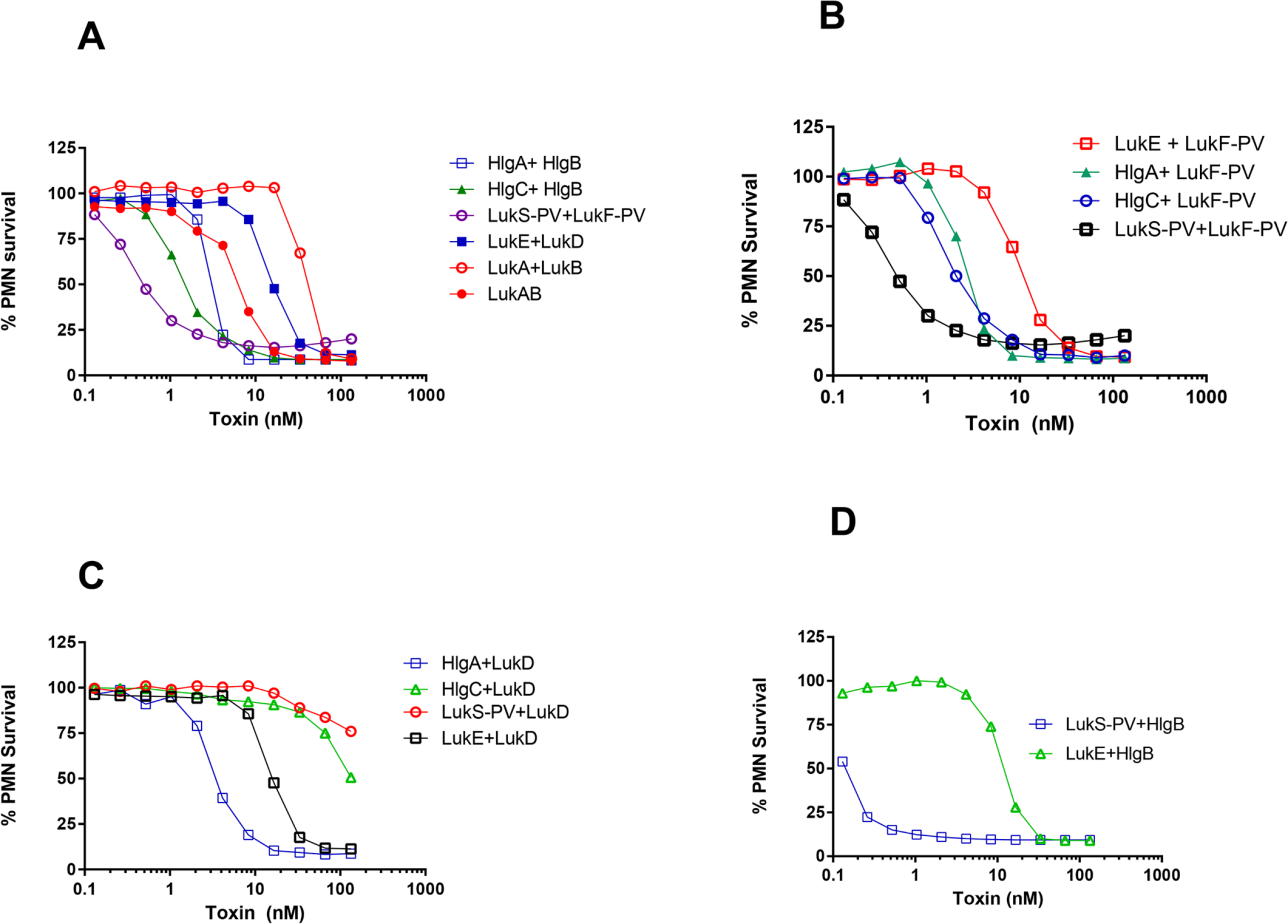
PMN lytic activity of different bicomponent toxins. Percentage (%) survival of HL-60 derived neutrophils treated with increasing concentrations of S-and F- component from different leukotoxin groups. ***A)*** Canonical pair ***B)*** Noncanonical pair with LukF-PV as F subunit ***C)*** Noncanonical pair with LukD as F subunit ***D)*** Noncanonical pair with HlgB as F subunit.

Pairs of leukotoxin subunits were tested in a cytotoxicity assay using HL-60 cells differentiated to neutrophils as we previously described [22, 25]. All five canonical pairs showed dose dependent cytotoxicity (**Fig 2A)**. Leukotoxins pairs LukS/F-PV (PVL) showed the highest toxicity with EC_50_ value of 0.4 nM followed by HlgCB and HlgAB with EC_50_ values ranging from 1–3 nM, whereas LukED and LukAB (mixture of two subunits) showed lower level of toxicity (**Table 1**) (**Fig 2A)**. When LukA and LukB were co-refolded as a complex, toxicity increased by at least 10 fold compared to the combination of the subunits refolded separately.

**Table 1:** PMN cytotoxicity of canonical and noncanonical lukotoxin subunit EC_50_ (nM).

S subunit			F subunit		
	LukF-PV	HlgB	LukD	LukB	Luk AB copurified
LukS-PV	0.4±0.16	0.1±0.03	>233.33	>133.3	
HlgA	1.7±0.96	1.5±0.86	3.6±1.2	>133.3	
LukE	11.5±4.3	11.26±3.1	15.4±2.9	>133.3	
HlgC	2.0±0.1	3.0±0.2	63.0±6.9	>133.3	
LukA	>133.3	>133.3	>133.3	108±2.1	
Luk AB copurified					8.1±0.96

Interestingly, mixing S and F subunits from different leukotoxin groups resulted in some toxin pairs (**Fig 2B–2D**) with EC_50_ values lower than canonical pairs (**Table 1**). The most potent toxin was created by pairing of LukS-PV with HlgB (EC_50_ = 0.1nM) (**Fig 2D** and **Table 1**). Combination of LukF-PV with HlgA or C also created potent toxins **(Fig 2B)**. When HlgA was paired with LukD it created a leukotoxin with 5 fold lower EC_50_ compared to LukED, while closely related HlgC paired with LukD was poorly cytotoxic (**Fig 2C** and **Table 1**). These data suggest that, if co-expressed *in vivo*, such non-canonical pairs can display significant toxicity and may contribute to pathogenicity.

### Rabbit anti-LukS-mut9 polyclonal cross-neutralizing antibodies

**Fig 3:**
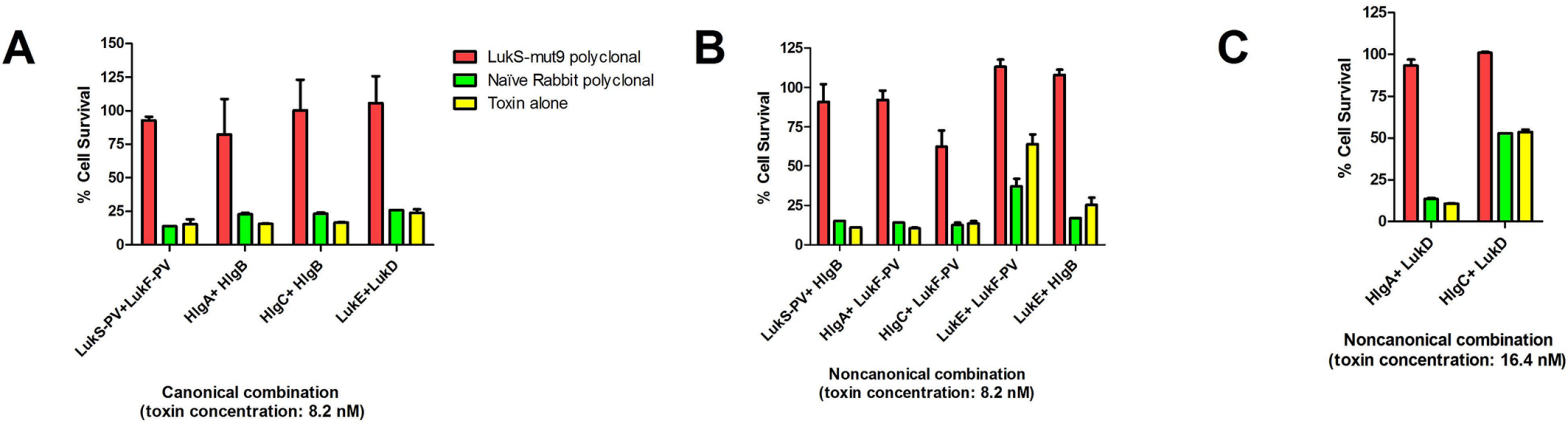
Rabbit anti-LukS-Mut9 antisera in 1:24 dilution neutralized PMN lytic activity induced by either canonical pairs or noncanonical pairs. Neutralization of: ***A)*** Canonical pair with 8.2 nM toxin concentration ***B)*** Noncanonical pair with 8.2 nM toxin concentration ***C)*** Noncanonical pair with 16.4 nM toxin concentration.

We examined LukS-Mut9 for its ability to generate cross neutralizing antibodies in rabbits. We analyzed the neutralization of canonical and non-canonical pairs using toxin concentration in the range of their EC_30_-EC_90_. Rabbit anti-LukS-mut9 (in 1:24 dilution) was able to effectively neutralize the canonical pairs PVL, HlgAB, HlgCB, and LukED **(Fig 3A)**, but not LukAB (data not shown). Polyclonal antibodies were also effective in neutralizing non-canonical pairs **(Fig 3B and 3C)**. These data clearly demonstrate the ability of antibodies raised against LukS-mut9 to broadly neutralize various leukotoxins.

### Hemolytic activities of canonical and non-canonical subunit pairs

**Fig 4:**
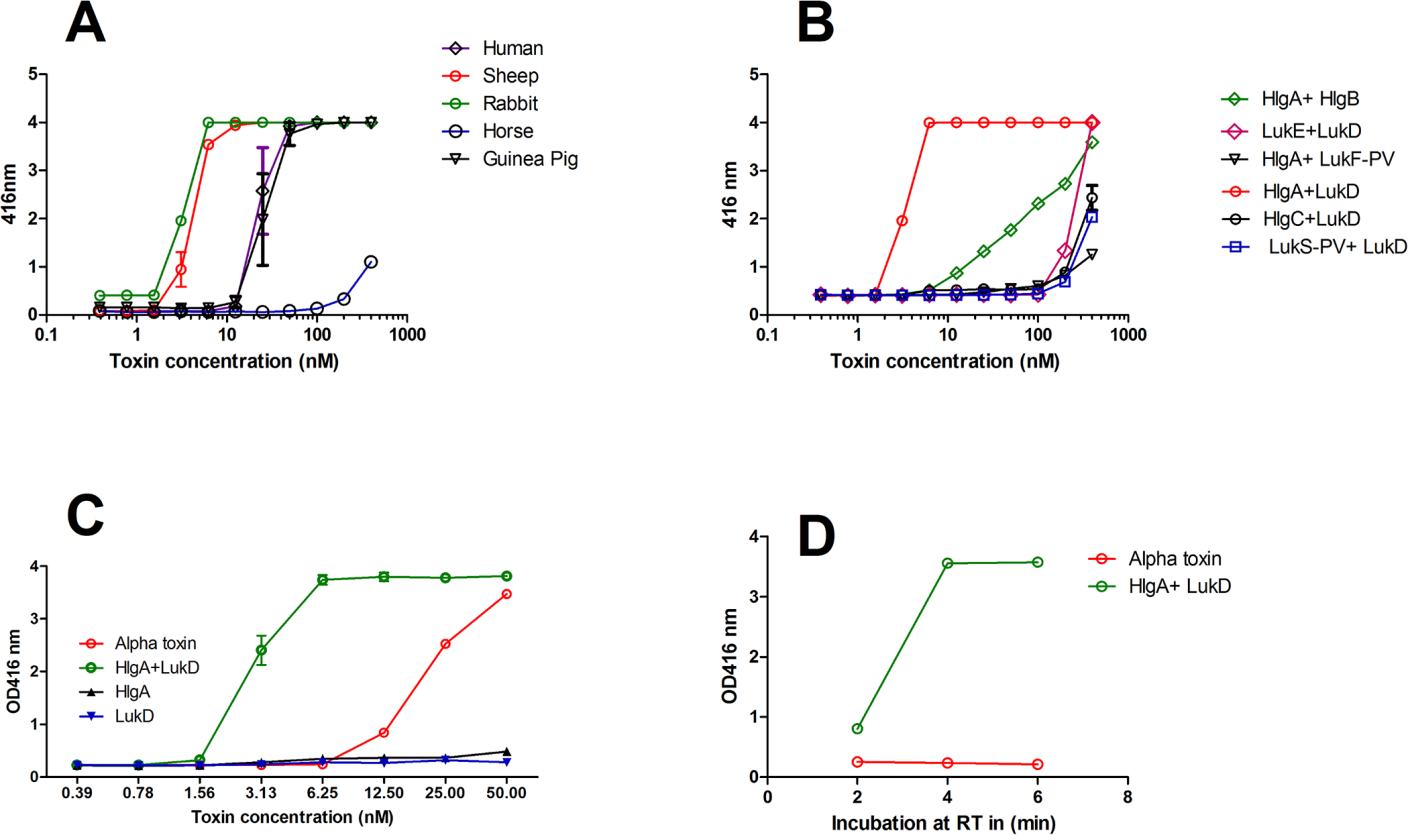
Noncanonical sub units: HlgA and LukD hemolysis studies. Lysis was measured at 416 nm. ***A***) Hemolytic dose response plot in 2% RBC (final concentration) from different species of blood. ***B***) Dose response plot in 2% rabbit RBC with all possible combination of HlgA and LukD showing that only HlgA + LukD makes a potent hemolytic pair. ***C***) Comparative dose response plot between alpha toxin and HlgA + LukD in 2% rabbit RBC. ***D***) Timed hemolysis between alpha toxin and HlgA + LukD in 2% rabbit RBC.

**Fig 5:**
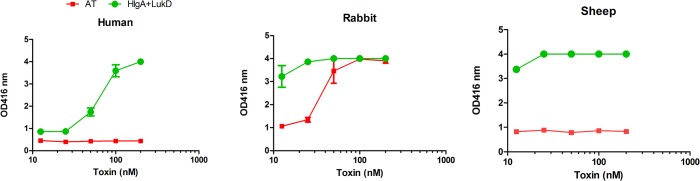
Comparative studies between alpha hemolysin and HlgA+ LukD in whole blood assay from different species. Blood from different species are indicated above the each panel. The assay was done in a whole blood (without washing with PBS) incubating with different concentration of toxin at 37°C for 30 or 45 min as descrided in method section.

There is limited published data available on the toxic activity of leukotoxins on RBCs from various species. In order to address this question, we examined the hemolytic activity of the BCPFTs towards human, mouse, rabbit, sheep, guinea pig, and horse RBCs. As expected most canonical and non-canonical pairs exhibited very low level of hemolysis in RBCs from different species (**Table 2**). However, certain non-canonical pairs especially HlgA/LukD showed high level of hemolysis in blood from multiple species. Notably, in contrast to alpha hemolysin (Hla), HlgA/LukD pair was strongly hemolytic towards rabbit RBCs **(Fig 4C).** In fact HlgA/LukD was hemolytic at EC50 values below 20nM in all species except horse **(Fig 4A, Table 2**). HlgAB was also hemolytic but far less than HlgA/LukD **(Fig 4B).** Either HlgA or LukD alone did not induce any hemolysis showing that both components are needed for this function **(Fig 4C).** Hemolysis induced by HlgA/LukD was extremely rapid, occurring within 2–4 minutes of exposure, while the kinetics of Hla induced hemolysis was much slower **(Fig 4D).** In order to address the question whether this noncanonical pair is active in whole blood, we titrated Hla and HlgA/LukD in whole blood from different species. In all three species tested, HlgA+LukD showed higher hemolysis than Hla **(Fig 5)**.

**Table 2:** Hemolysis (EC_50_) for different canonical and noncanonical leukotoxins pairs to different species of RBCs.

S-Subunit	F-Subunit			EC_50_ (nM)			
		Human	Sheep	Rabbit	Guinea Pig	Mouse	Horse
	LukF-PV	>200	>200	>200	>200	>200	>200
LukS-PV	HlgB	>200	>200	>200	>200	>200	>200
	LukD	>200	>200	>200	>200	>200	>200
	LukF-PV	>200	>200	>200	>200	>200	>200
HlgC	HlgB	>200	>200	>200	>200	>200	>200
	LukD	>200	>200	>200	>200	>200	>200
	LukF-PV	>200	>200	>200	>200	>200	>200
HlgA	HlgB	>200	>200	79±21.9	>200	>200	>200
	LukD	11.5±2.6	2.04±0.21	1.62±0.02	13.3±8.13	11.35±0.07	>200
	LukF-PV	>200	>200	>200	>200	>200	>200
LukE	HlgB	>200	>200	>200	>200	>200	>200
	LukD	111.2±0.35	82±0.7	122.5±1.4	>200	>200	>200
Alpha toxin	-	>200	70±2.1	3.7±0.07	>200	20±1.5	>200

**Fig 6:**
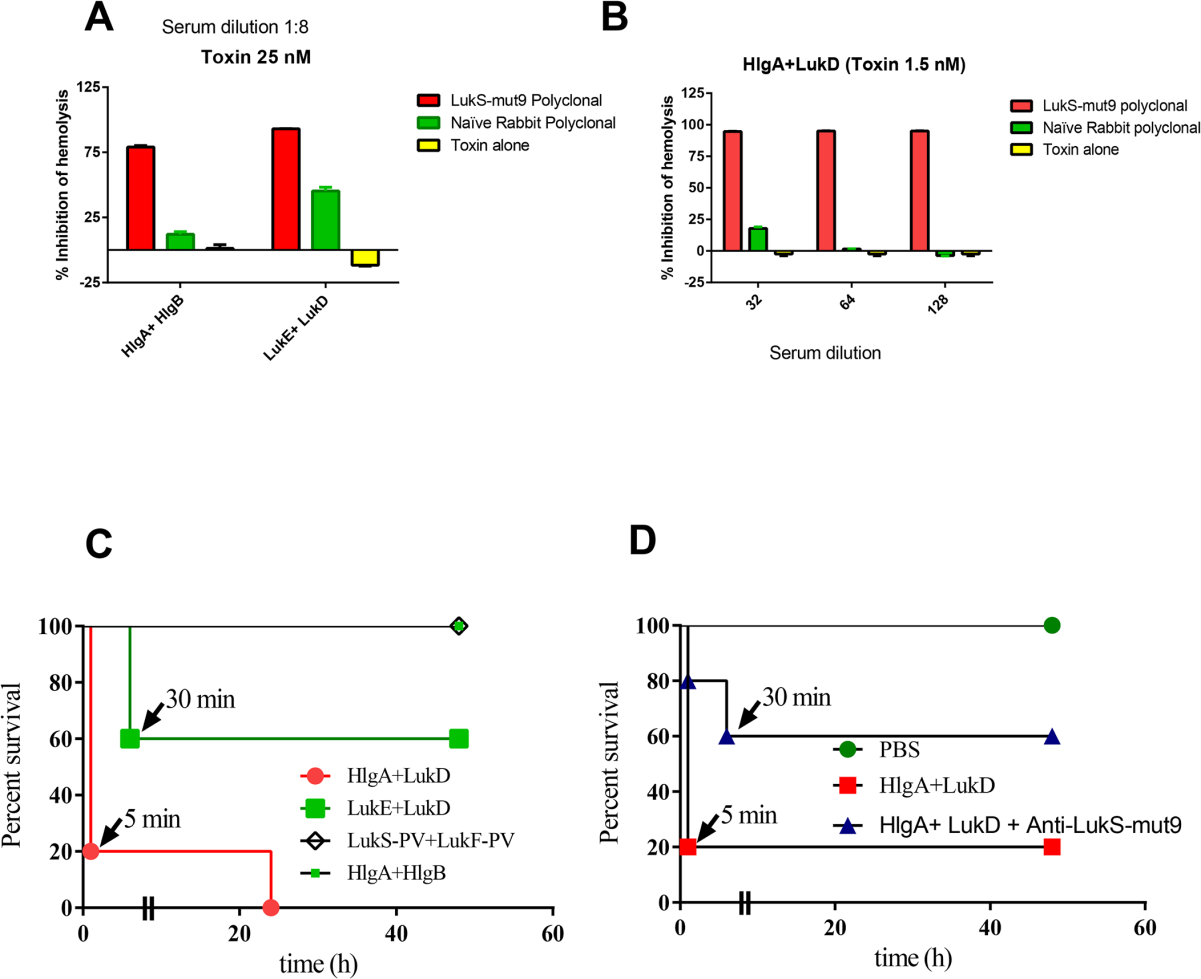
Rabbit anti-LukS-Mut9 antisera neutralized RBC lytic activity induced by either canonical pairs or noncanonical pairs. ***A)*** Neutralization Canonical pair ***B)*** Neutralization of noncanonical pair (HlgA+LukD at 1.5 nM) with diluted serum samples. **C)** Determination of comparative lethality between different bicomponent toxins. Five ICR mice were challenged through IV route with 25μg/mouse of the following: HlgA+LukD, LukE+LukD, LukS-PV+LukF-PV and HlgA+HlgB. LukS-PV and LukF-PV were used as control leukotoxin. Mice were monitored every minute for first hour and then every day for 3 days for lethality. **D)** Protective efficacy of anti-LukS-mut9 polyclonals. Five Balb/c mice were challenged through IV route with 10 μg/mouse of HlgA+LukD. For rescue study, 750ug/mouse total antibodies were passively immunized through IV route before 5 minutes of toxin challenge. Mice were monitored every minute for first hour and then every day for 3 days for lethality.

For those pairs that showed clear hemolysis in rabbit whole blood, toxin neutralization was tested using rabbit anti-LukS-mut9 polyclonal serum. With 1:8 dilution of the rabbit anti-LukS-mut9 polyclonal serum, we observed 80% and 90% inhibition of hemolysis induced by 25nM of HlgAB and LukED respectively (**Fig 6A**). The neutralizing titer of anti-LukS-mut9 against HlgA/LukD was higher with >95% neutralization at 1:128 dilution when the toxin was used at 1.5nM (**Fig 6B**).

### *In vivo* toxicity of leukotoxins

To examine if the canonical and non-canonical pairs exert severe toxicity *in vivo*, we evaluated the acute toxicity of HlgA/LukD, HlgAB, LukED, and PVL in ICR mice. Groups of 5 mice were challenged with 25 μg/mouse total toxin by IV route. As shown in **Fig 6C,** HlgA/LukD killed 80% of mice within 5 minutes of toxin administrations and LukED showed 40% lethality. In contrast, all mice treated with HlgAB or PVL survived the challenge. As the lethality in HlgA and LukD group was very high and the death occurred extremely rapidly, we measured the endotoxins level of these toxins to rule out the possible contributions of endotoxin in lethality. Endotoxin levels were low for both sub units. The values for HlgA and LukD were the 3.3 and 6.3 Endotoxin Units/mg respectively.

### Efficacy of anti-LukS-Mut9 in HlgA and LukD challenged Mice

We also carried out HlgA/LukD rescue study by using Polyclonal anti-LukS-mut9 in Balb/C mice. Groups of 5 mice were challenged with 10 μg/mouse total toxin by IV route. For this experiment, we purified the total antibodies from crude serum samples by protein “A” purification column. As shown in **Fig 6D**, 10ug/mouse of toxin (HlgA + LukD) killed 80% of the mice within 5 minutes, whereas when mice were passively immunized with 750 ug of anti-lukS-mut9 polyclonal (total antibody) 5 minutes before challenge, 60% of the mice survived the challenge with one death occurring in the first 2 minute and second died after 30^th^ minute of challenge.

### Association between IgG antibody level to the different Leukotoxin subunits and sepsis

**Fig 7:**
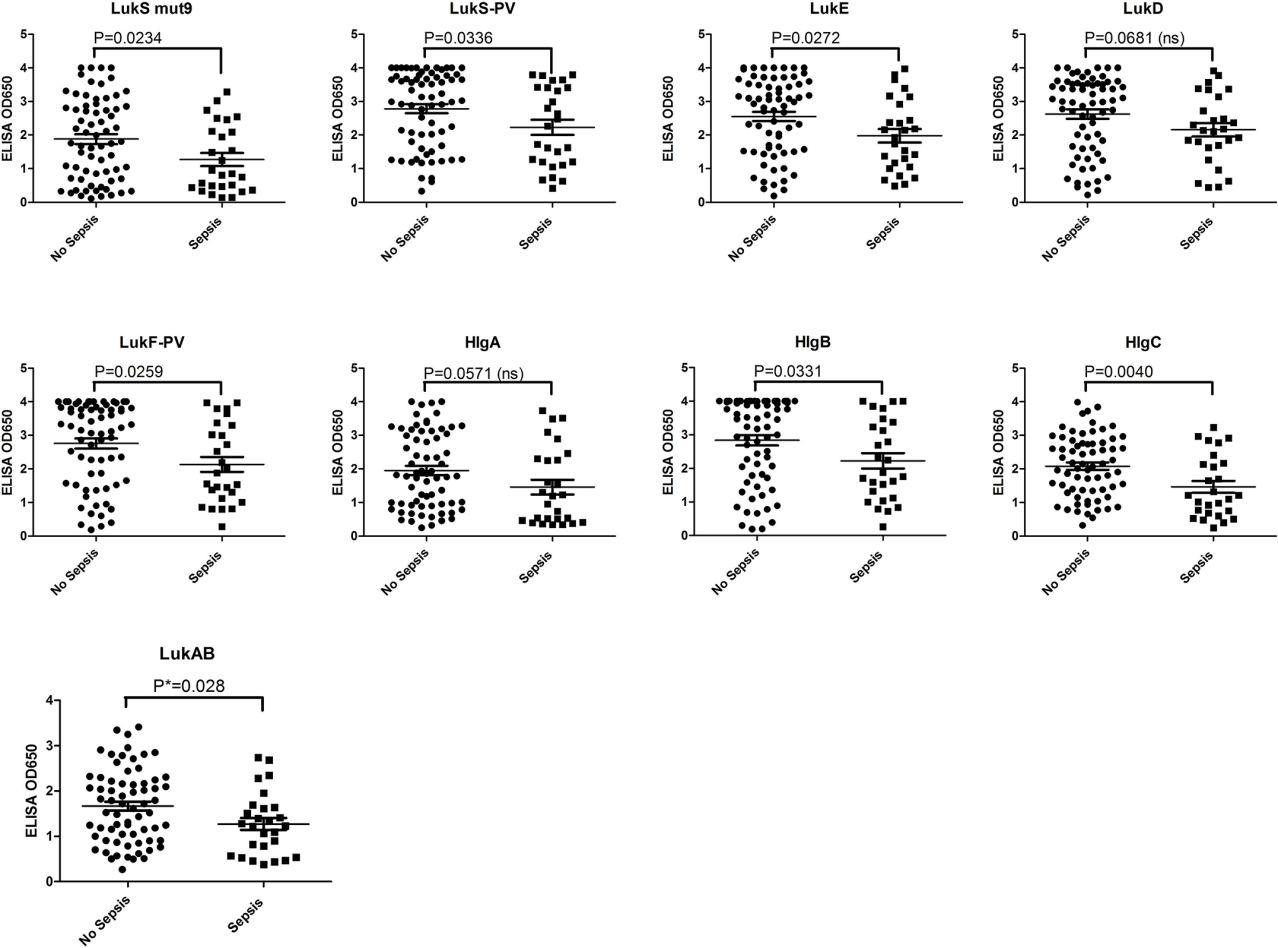
ELISA of clinical samples classified into sepsis and nonsepsis based on clinical outcome. Individual toxin subunits as indicated above the each panel were coated over night at 4°C. Human serum samples were diluted to 1:10,000 in PBS with 1% milk. Goat anti human HRP conjugate diluted to 1:2,000 was used as secondary antibody.

We recently reported an inverse correlation between the probability of sepsis in adult patients with SA bacteremia and preexisting antibody titers to several staphylococcal major toxins including LukS-PV and LukF-PV [26]. To expand this study, we used the remaining serum samples from these patients to evaluate the reactivity of the sera against purified leukotoxin subunits by ELISA.LukS-PV and LukF-PV were used as positive controls. As shown in **Fig 7**, the reactivity of the sera to LukS-PV, LukF-PV, LukE, HlgB, HlgC, LukAB, and LukS-mut9 was significantly lower in patients with sepsis outcome than in patients who did not develop sepsis. While a trend towards lower antibody titers in septic patients was observed for HlgA and LukD, the difference was not statistically significant as tested by student's unpaired t-test (**Fig 7**). The highest degree of correlation was observed for LukD, LukF, LukS-mut9, and LukAB and HlgC.

## Discussion

*S*. *aureus* has grown increasingly antibiotic resistant over the past few decades and causes a number of diseases ranging from superficial skin infections to life threatening endocarditis and septicemia. Studies have shown that about 33% of the population carries *S*. *aureus* and 2% carries MRSA (http://www.cdc.gov/mrsa/healthcare/). Although the rate of nosocomial severe MRSA infections in the United States is declining, MRSA still remains an important public health problem. According to the 2012 CDC report, PVL-positive strain USA300 caused more than 69% of the CA-MRSA and 34.6% of HA-MRSA cases and PVL-negative USA strains like: USA100 and USA500 are responsible the remaining cases. These PVL negative strains can still produce a number of other bicomponent leukotoxins, which have high level of homology in protein structural as well as in amino acid sequences to PVL subunits, suggesting that they may have overlapping functions.

Bicomponent leukotoxins are a major group of toxins produced by staphylococci. Their toxic effects depend on the synergistic action of two proteins, and single components are either nontoxic or exert very little toxicity. One subunit is known as S (e.g. LukS-PV, LukA, HlgA, HlgC, LukE) and the second subunit referred to as F (e.g. LukF-PV, LukB, HlgB, LukD) [16, 29] [30]. The S subunit typically mediates receptor binding and is the first component to interact with the membrane [31]. Binding of S subunit triggers a process in which an octamer consisting of alternately arranged S and F subunits is formed leading to pore formation in the host cell membranes. While the term leukotoxin was originally coined based on toxic effect on leukocytes, these leukocidins are cytotoxic towards a variety of cells types [23] including human polymorphonuclear leukocytes (PMNs) [32], monocytes and macrophages [33, 34].

The role of PVL in the pathogenesis of CA-MRSA has been controversial [35, 36]. A study using PVL-positive and PVL-negative CA-MRSA showed that isogenic PVL-negative (LukS/F-PV knockout) strains of USA300 and USA400 were as lethal as wild-type strains in mouse sepsis and skin infection models [37]. In contrast, several other reports indicated that PVL plays an important role in pathogenesis of staphylococcal disease in different animal models [38–41]. Our recent report showed that pre-existing antibodies to PVL confers protective advantage against sepsis in *S*. *aureus* bacteremic patients [26]. In another report, we showed the efficacy of LukS-mut9, an attenuated subunit vaccine for *S*. *aureus* LukS-PV in a mouse bacteremia model [22]. Since mice do not have a receptor for PVL [42], the observed protective efficacy of attenuated LukS vaccine is most likely related to cross reactivity with other leukotoxins that are toxic in mice [22].

While PVL has been most extensively studied, a number of recent reports clearly indicate a range of unique and overlapping functions for several members of the BCPFT family. Gamma hemolysins, expressed in nearly all *S*. *aureus* isolates [43] [44], play a role in *S*. *aureus* survival in blood [45], and in the pathogenesis of septic arthritis [46]. LukED plays a key role in *S*. *aureus* bloodstream infection [19]. In contrast to PVL, LukED is expressed by 60–70% of clinical isolates [43]. LukAB is less than 30% [23] homologous to other BCPFTs, but appears to play a major role in *S*. *aureus* virulence[47]. Thus, *S*. *aureus* is equipped with a variety of BCPFTs to exert cytotoxic and modulatory effects on the innate immune responses. Furthermore, several previous reports demonstrated the ability of individual BCPFT subunits to pair in a non-canonical fashion to form functional toxins [23, 24]. It remains to be determined if non-canonically paired BCPFTs play a role in the pathogenesis of *S*. *aureus* infection.

Cellular tropism and species specificity of the BCPFTs are regulated by specific receptors. PVL utilizes human complement receptors C5aR and C5L2 and is strictly cytotoxic towards human and rabbit cells [42] [21], while LukED interacts with both human and mouse chemokine receptors CCR5, CXCR1, and CXCR2 [8]. Spaan *et al* [42] identified three specific receptors, CXCR1, CXCR2, and CCR2 for HlgAB, and two receptors, C5aR and C5L2 for HlgCB. Based on these receptor expression profile, cells are susceptible to either or both HlgAB and HlgCB which help the toxins to efficiently and differentially target phagocytic cells [42]. In light of these findings showing lack of functional receptors for PVL in mice, previous reports [22, 48] demonstrating protective activity of anti-PVL antibodies in mice must relate to cross protection against other leukotoxins that are functional in the murine system. To this end, we sought to examine the breadth of neutralizing activity of antibodies raised against an attenuated LukS-PV mutant (T28F/K97A/S209A; LukS-mut9) [22] against all major BCPFTs. Our findings demonstrate that anti-LukS-mut9 antibodies effectively neutralize PVL, HlgAB, HlgCB, and LukED, suggesting that this mutant can be an important component of a multivalent *S*. *aureus* vaccine. In contrast, no significant neutralization of LukAB was afforded by anti-LukSmut9 antibodies, consistent with low sequence homology of this toxin with other BCPFTs. Thus for a complete coverage of all BCPFTs by multivalent vaccine addition of an attenuated LukAB toxoid may be required.

Previous reports have indicated non-canonical pairing of BCPFTs. Studies in rabbits with five different canonical and noncanonical combinations of S and F subunits from PVL (LukS-PV and LukF-PV) and Hlg (HlgA, B and C) [49] showed various degrees of toxicity. The reported order of severity of symptoms based on inflammation and necrosis was: HlgA+LukF-PV>HlgAB≥LukS-PV+HlgB≥PVL>HlgCB. Combination of HlgA and LukF-PV has been shown to be hemolytic towards rabbit red blood cells [50]. Recently, we have reported that HlgB can form non-canonical pairs with LukS-PV [22]. Here, we further investigated the spectrum of cytotoxic activities that can be afforded by non-canonical pairing of BCPFT subunits. Strikingly, combination of LukS-PV with HlgB created the most potent toxin among all BCPFTs being 4–5 times more toxic than PVL, 30 times more toxic than HlgCB, and 15 times more toxic than HlgAB towards human PMN **(Table 1)**. HlgA, paired with LukF-PV, was as toxic as its canonical form of HlgAB. HlgA, paired with LukD, also generated a toxin with five-fold higher toxicity than LukED. This was highly specific to HlgA, since HlgC paired with LukD was four times less toxic than LukED. We also investigated the breadth of hemolytic activities of all possible BCPFT combinations towards RBCs from six different species. As expected most canonical and non-canonical BCPFTs lacked appreciable hemolytic activity (**Table 2**). As the only canonical BCPFTs with hemolytic activity, HlgAB lysed rabbit RBCs with an EC_50_ of 79 nM, and LukED lysed RBCs from humans, sheep, and rabbits with EC_50_ values of 111, 82, and 122 nM, respectively. These high EC50 values are most likely physiologically not relevant. However, non-canonical pairing of HlgA with LukD generated a highly hemolytic toxin that rivals alpha hemolysin. HlgA/LukD lysed RBC from human, sheep, rabbit, guinea pigs and mice with EC_50_ of 11.5, 2, 1.6, 13.3, and 11.3 nM, respectively. Consistent with the high toxicity of HlgA/LukD towards both RBCs and PMNs, this non-canonical pair was acutely toxic in mice and killed 100% of mice with five minutes to 24 hours **(Fig 6C)**. Also LukED, known to target murine cells was highly toxic in mice, while PVL or HlgAB showed no acute toxicity *in vivo*. This study also generated a proof of concept data for the protective efficacy of LukS-mut9 polyclonal **(Fig 6D)** against HlgA+ LukD toxicity in mice rescue study.

Our findings indicate that antibodies against LukS-mut9 effectively neutralize the cytotoxicity of non-canonical BCPFT pairs in both PMN **(Fig 3)** and hemolytic **(Fig 6)** assays. While, the pathophysiological relevance of non-canonical BCPFTs remains to be determined, these data suggest that, if co-expressed *in vivo*, such pairings can have major implications for *S*. *aureus* pathogenicity. Future studies should evaluate this possibility.

In this report, we show that LukAB toxicity is >10 times higher when both subunits are co-refolded together (**Table 1**), consistent with a recent report that LukAB exists as a heterodimer in solution rather than two separate monomers [28]. However, the fact that LukA and LukB can also be separately refolded in monomeric form and show activity when combined is important from the vantage point of vaccine development. Isolated LukA or LukB are not likely to be toxic and can be used to induce neutralizing antibodies without imposing a safety risk to vaccinated individuals. Alternatively, an attenuated LukA or LukB vaccines can be rationally designed to further mitigate safety concerns.

In our previous report, we showed a significant inverse correlation between sepsis and specific antibody titer against Hla, LukS-PV and delta toxin in adult patients with *S*. *aureus* bacteremia [26]. In the current report we expanded these studies to examine the correlation between antibody response to all BCPFTs and sepsis using the same serum samples. Our findings indicate that antibody titers to LukS-PV, LukF-PV, LukE, HlgB, HlgC, and LukAB significantly and inversely correlate with septic outcome of infection (**Fig 7**). The correlation for HlgA and LukD was not statistically significant (P values of 0.057 and 0.068, respectively) although a trend in the same direction could be observed. A limitation of these data is that a single dilution of the sera was tested in ELISA because of the limited availability of the sera. Future studies should clarify the role of such antibodies in the outcome of *S*. *aureus* in bacteremic patients. Importantly, our data show that antibodies reactive to LukS-mut9 vaccine candidate show significant correlation with lack of sepsis (P = 0.0234). This finding further suggests that this attenuated toxoid is able to induce relevant antibodies if used as a vaccine.

In summary, this report shows that LukS-mut9, a rationally designed LukS-PV toxoid vaccine, can elicit a broadly neutralizing polyclonal response against bicomponent pore forming toxins. Thus, a LukS-mut9 based vaccine is able to provide a broad spectrum of activity against leukotoxic and hemolytic bicomponent toxins. Our data also indicated that for a complete coverage of all BCPFTs, an additional vaccine component consisting of either LukA or LukB is required.

## Supporting Information

S1 Checklist NC3Rs ARRIVE Guidelines. (PDF)
