# Correction: Antibodies to *S*. *aureus* LukS-PV Attenuated Subunit Vaccine Neutralize a Broad Spectrum of Canonical and Non-Canonical Bicomponent Leukotoxin Pairs

**DOI:** 10.1371/journal.pone.0143493

**Published:** 2015-11-16

**Authors:** Rajan P. Adhikari, Thomas Kort, Sergey Shulenin, Tulasikumari Kanipakala, Nader Ganjbaksh, Mary-Claire Roghmann, Frederick W. Holtsberg, M. Javad Aman

There is an error in the first sentence of the Funding section. The first sentence should read: This work was partially supported by grants (AI106162, AI098232 to R.P.A and AI111205 to M.J.A) from the National Institute of Allergy and Infectious Diseases (NIAID) and partially from Integrated BioTherapeutics, Inc. (IBT) internal R&D research fund.
